# Place of magistral preparations to continue the treatment if the drug is commercially stopped worldwide? A case report of a 10-year-old child with subacute sclerosing panencephalitis (SSPE) requiring inosiplex

**DOI:** 10.1080/22221751.2022.2148563

**Published:** 2022-12-20

**Authors:** Renan Le Cras, Roseline Mazet, Fanny Dubois-Teklali, Cécile Sabourdy, Sébastien Chanoine, Audrey Lehmann, Agathe Morin, Julien Leenhardt, Marjorie Durand, Marie-Dominique Desruet, Pierrick Bedouch

**Affiliations:** a Pharmacy Department, Grenoble University Hospital, Grenoble, France; b Pediatric Neurology Unit, Grenoble University Hospital, Grenoble, France; c Neurology Unit, Grenoble University Hospital, Grenoble, France; d Université Grenoble Alpes TIMC, CNRS UMR 5525, Grenoble, France; e Université Grenoble Alpes, INSERM, LRB, Grenoble, France

**Keywords:** Inosiplex, inosine pranobex, speciality commercially stopped, magistral preparation, subacute sclerosing panencephalitis (SSPE)

## Abstract

Subacute sclerosing panencephalitis (SSPE) is a late-onset and fatal viral disease caused by persistent infection of the central nervous system by measles virus (MeV). We present the case of a 10-year-old child from South Asia affected by SSPE, stabilized with a combination of intrathecal interferon-α2b (INF-α2b) injections and oral inosiplex and how we continued the treatment when inosiplex was commercially stopped worldwide.

## Introduction

Measles is an extremely contagious vaccine-preventable disease caused by the measles virus (MeV), belonging to *Paramyxoviridae* family. The illness typically begins with fever and pathognomonic enanthem. Complications occur in 10–40% of cases and subacute sclerosing panencephalitis (SSPE) is one of the most dramatic [1]. The highest SSPE incidence is estimated at 360/100,000 measles cases in the Middle East [2]. With widespread measles vaccination, measles diseases and complications have declined. Unfortunately, large epidemics are still present due to conflicts, population gatherings, and lack of health infrastructure in some geographical areas [3].

SSPE is a progressive neurological disease associated with high mortality within 1–3 years of onset [2–6]. Patients develop severe mental and physical impairments, with loss of motor control that tends to evolve in myoclonic jerks, spasms, seizures, dementia, and coma [7]. SSPE is usually starting 8–11 years after measles disease. Its pathogenesis is not yet fully understood but it may be caused by a persistence of mutated measles virus (MeV) in the central nervous system (CNS), leading to demyelinating lesions [5].

SSPE treatments include antiviral agents such as isoprinosine, interferon-alpha, or ribavirin. These drugs are usually associated with anticonvulsants such as carbamazepine, levetiracetam, and clobazam to control myoclonic jerks. In the future, a fusion inhibitory peptide may be used [5]. Currently, inosiplex in combination with weekly intrathecal interferon-α (INF-α) is considered most beneficial to treat SSPE [7,8]. Nevertheless, they are rarely been shown to recover loss of function, but they can stabilize the disease for several years [9]. Inosiplex or inosine pranobex is a blend of three molecules: inosine, acetamidobenzoïc acid, and dimethylaminoisopropanol, commercially named ISOPRINOSINE^®^ [2,8]. Despite the advantage of INF-α2b remains unclear, combination therapy is still used because of potential synergistic effects [8,9]. However, no treatment can cure the disease [2,7,9].

Our work aims to present the case of a 10-year-old child affected by SSPE who was stabilized with a combination of intrathecal INF-α2b injections and oral inosiplex and how we continue the treatment when ISOPRINOSINE^®^ 500 mg tablets (Sanofi Aventis, France) were commercially stopped. This illustrates the real public health problem related to a supply disruption or a marketing cessation of a pharmaceutical specialty, its impact on the clinical management of patients, and the essential role of hospital pharmacotechnical units.

## Case

The patient was born in Afghanistan, without any complications. He contracted measles at 6 months old without any complications. No vaccination was recorded. Unfortunately, in December 2017, he was hospitalized due to a brutal neurological degradation, with central hypotonia, spastic tetraplegia, fever flares, and generalized tonic-clonic seizures. The diagnosis of SSPE was made in India in June 2018 with an elevated level of anti-measles antibodies in cerebrospinal fluid and serum. Electroencephalography (EEG) finds asymmetric, unorganized, and slowed-down tracings, with rapid periodic bursts. In addition, magnetic resonance imaging (MRI) showed lesions consistent with SSPE. Intrathecal injection of IFN-α2b RELIFERON® (Reliance life sciences) 3.10^6^ units/m^2^/week was initiated, associated with ISOPRINOSINE® 500 mg tablets (New Port Pharmaceutical Limited) 33 mg/kg/8 h. Seizures were treated with usual antiepileptic drugs (Supplementary Figure S1).

On his arrival in France in January 2019, the patient presented spastic stiffness and almost no motricity of the four limbs with hyperreflexia on the left side. The Babinsky’s sign was uncertain. He had clonic eye movements and no ocular following. The pupillary reflex was normal.

**Figure 1.**
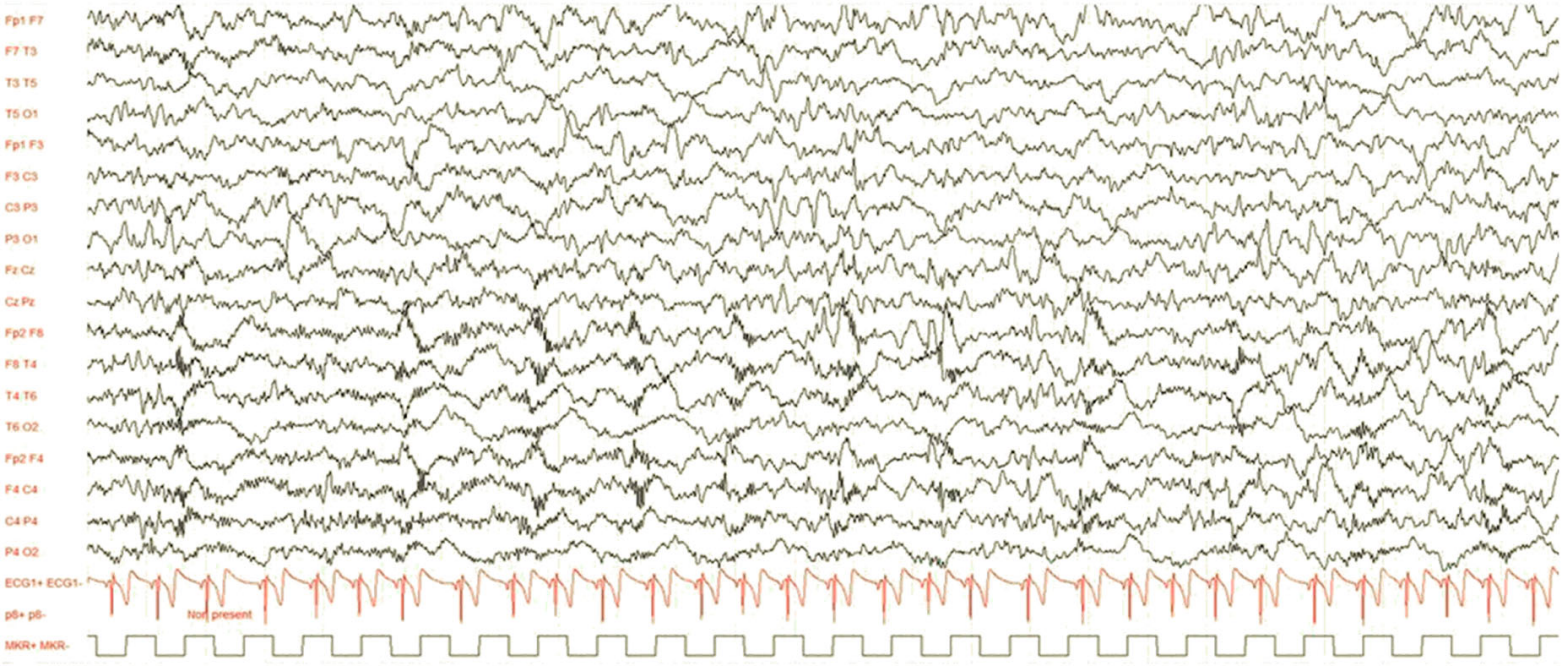
Patient extended EEG from June 2019.

Figure 1 presents an extended EEG showing wide and irregular waves in the *θ* range (5–6 Hz), with a left-right asymmetry. High-frequency, pseudo-periodic, fronto-central bursts were seen. Sleep amplified the previously described abnormalities.

**Figure 2.**
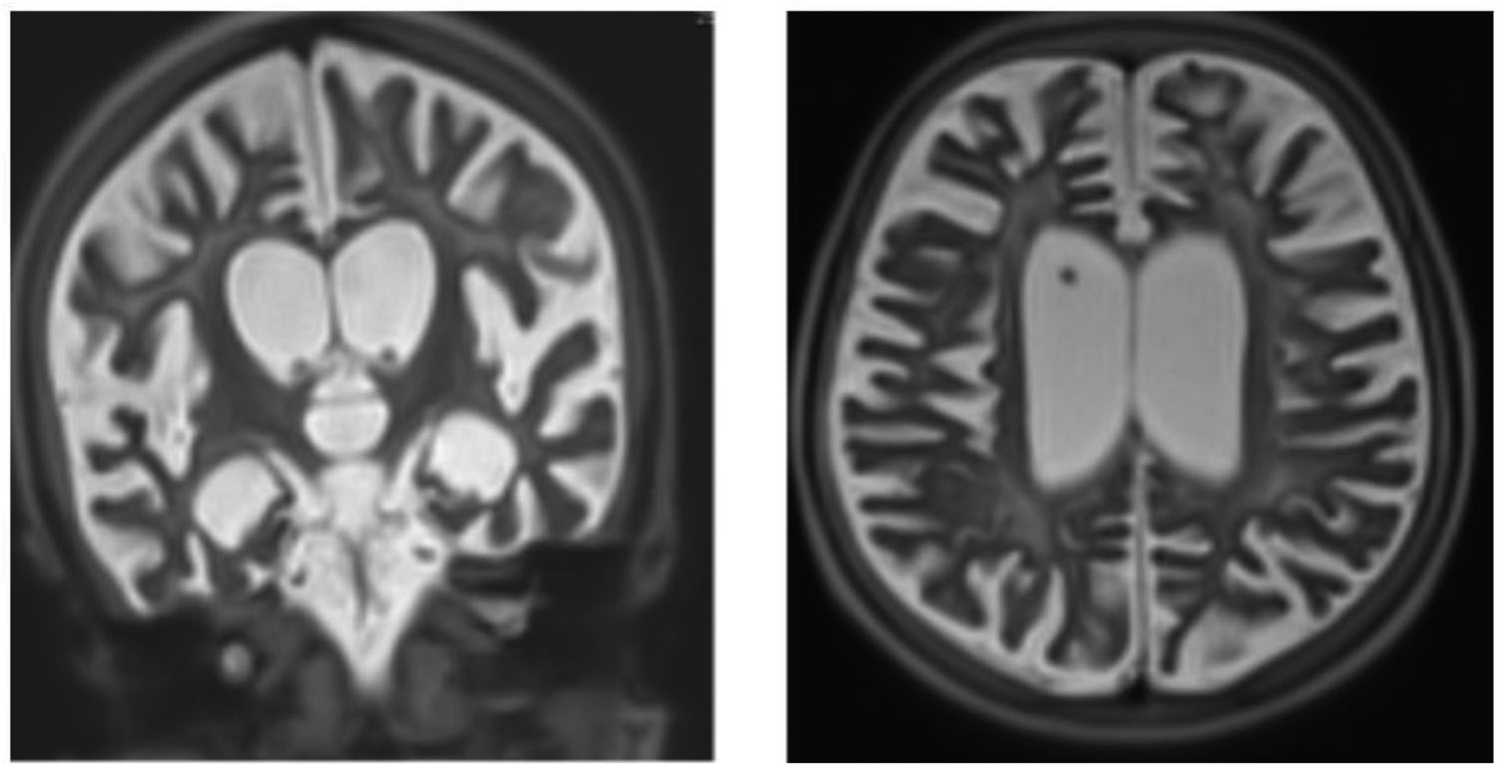
MRIb from June 2019 shows cortical and subcortical atrophy.

A brain magnetic resonance imaging (MRIb) showed cortical and subcortical atrophy, with ventricular and peri-cerebral enlargement. There were diffuse signal anomalies of the white matter, with a hyperintensity in the T2 sequence and hypointensity in the T1 sequence. Basal ganglia and corpus callosum were atrophic and hyperintensity was seen in the internal capsule. In the subtentorial region, there was a demyelination aspect of both mesencephalon and metencephalon. (Figure 2).

Chemiluminescence assay on the LCR showed a high anti-MeV IgG antibody titre (3330 UAI/mL; positive >16.5 UAI/mL). Serology was also positive for anti-MeV Immunoglobulin G (IgG). Polymerase chain reaction and all serologies were negative for neurotropic viruses, *Mycoplasma pneumoniae*, *Bartonella* sp., and *Toxoplasma gondii*. Serologies for *Cytomegalovirus* and *Epstein–Barr virus* showed a high level of IgG.

Decision was made by a multidisciplinary committee to continue the treatment with IFN-α2b and inosiplex [10], combined with levetiracetam (20 mg/kg/day), sodium valproate (14.4 mg/kg/day), clonazepam (0.02 mg/kg/day), and zonisamide (3.2 mg/kg/day) to treat tonic–clonic seizures.

Concerning IFN-α2b, it was initially administered at the dose of 3.10^6^ units/m2 per week during a month, then the maintenance dose was continued at 3.10^6^ units/m^2^ per month. He received only six injections of IFN-α2b because INTRONA® was discontinued in France, and supply became impossible in March 2020.

The paediatric unit requested inosiplex in June 2019. Unfortunately, only SANOFI AVENTIS marketed, in Europe, a speciality containing inosiplex (ISOPRINOSINE^®^ 500 mg) and stopped the worldwide marketing in 2014 due to low sales volume. After 3 months of research, the unit for medicines under temporary use authorization in Grenoble University Hospital (CHUGA) was able to import ISOPRINOSINE^®^ 500 mg (Sanofi Aventis) from Belgium. The patient received therefore 20 mg/kg/8 h. Due to his clinical situation, the French drug agency authorized the importation of the specialty and Sanofi Aventis France has graciously offered ISOPRINOSINE^®^ for this compassionate use. As of October 31, 2021, all remaining Sanofi Aventis stock expired. Given the lack of any alternative to provide for this patient, it was considered to make a magistral preparation. We looked for a Good Manufacturing Practices (GMP) supplier of this active pharmaceutical ingredient. In Europe, only one supplier was identified and with the precious help of MedicaPharma (Netherlands), we prepared 500 mg hard capsules of inosiplex in the preparation unit of CHUGA. The patient continued the treatment on an outpatient basis from November 18, 2021 (Supplementary Figure S1).

During the 18 days of treatment’s interruption, the patient's clinical condition deteriorated with an increase in the frequency of seizures. Caregivers reported that his condition had gradually improved since the reintroduction of inosiplex.

Today, the patient's health condition is stabilized. Moreover, his neurological disorders, in particular dysautonomia, have not increased and no new tonic-clonic seizures were reported.

## Conclusion

Inosiplex preparation has contributed to stabilize his health condition until today. It was the only way to provide a safe response to the patient in this situation. The main challenge was to supply raw material for pharmaceutical use at a GMP grade, as inosiplex has a very limited indication and low frequency of use in Western countries. Thus, to our knowledge, CHUGA is the only hospital in France to provide this treatment by inosiplex. This case illustrates the problem of pursuing a treatment when a speciality is no longer marketed, reinforcing the interest in magistral preparation and the role of hospital pharmacies in the current context where stock outs are more and more frequent.

## Supplementary Material

Supplemental Material
