# Supplementary figures and images for: Place of magistral preparations to continue the treatment if the drug is commercially stopped worldwide? A case report of a 10-year-old child with subacute sclerosing panencephalitis (SSPE) requiring inosiplex
Source: Emerg Microbes Infect. 2022 Dec 20;12(1):2148563. doi: 10.1080/22221751.2022.2148563 (PMC9788679; doi:10.1080/22221751.2022.2148563)

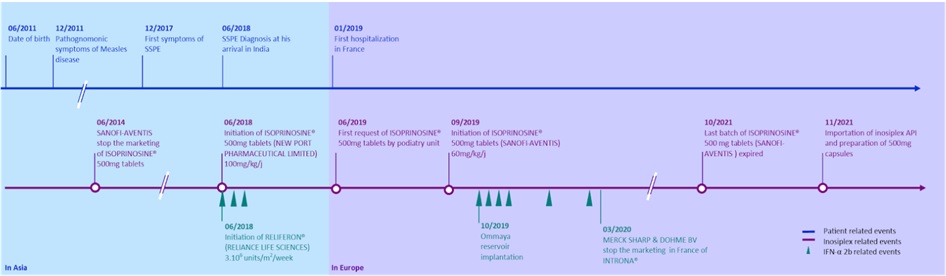

Supplement: Supplemental Material [file TEMI_A_2148563_SM6095.jpg]
